# Differences in Artificial Intelligence-Based Macular Fluid Parameters Between Clinical Stages of Diabetic Macular Edema and Their Relationship with Visual Acuity

**DOI:** 10.3390/jcm14031007

**Published:** 2025-02-05

**Authors:** Mizuho Mitamura, Michiyuki Saito, Kiriko Hirooka, Zhenyu Dong, Ryo Ando, Satoru Kase, Susumu Ishida

**Affiliations:** Department of Ophthalmology, Faculty of Medicine and Graduate School of Medicine, Hokkaido University, N-15, W-7, Kita-ku, Sapporo 060-8638, Japanishidasu@med.hokudai.ac.jp (S.I.)

**Keywords:** diabetic macular edema, deep learning, imaging, semantic segmentation

## Abstract

**Background/Objectives**: The aim of this study was to determine artificial intelligence-based macular fluid (MF) parameters in diabetic macular edema (DME) with optical coherence tomography (OCT) and examine stage-by-stage differences in MF parameters and their relationship with best-corrected visual acuity (BCVA). **Methods**: This study enrolled 104 eyes with treatment-naïve DME. Intraretinal fluid (IRF) and subretinal fluid (SRF) were detected in horizontal OCT images based on the “Hokkaido University MF segmentation model” when DME was first observed together with BCVA testing. The MF area, the mean brightness, and the variance of brightness were compared between mild or moderate non-proliferative diabetic retinopathy (mNPDR, n = 33), severe NPDR (sNPDR, n = 52), and PDR eyes (n = 19). Correlations between logMAR BCVA and MF parameters were also examined. **Results**: All the MF parameters tended to increase with DR stages. Especially, the mean brightness of IRF was significantly greater in PDR than in mNPDR. The variance of brightness of IRF increased in sNPDR compared to mNPDR, whereas that of SRF increased in PDR compared to sNPDR. LogMAR BCVA showed positive correlations with MF areas and the variance of brightness of SRF. **Conclusions**: The qualitative and quantitative MF parameters may be useful for better understanding DME pathogenesis according to DR progression.

## 1. Introduction

Diabetic macular edema (DME) is one of the major threats to sight in diabetic retinopathy (DR) [1]. Various modalities have been used as indicators for DME treatment, and, in particular, optical coherence tomography (OCT) provides ophthalmologists with a variety of information about DME such as intraretinal fluid (IRF), subretinal fluid (SRF), and retinal thickness, and is an indispensable tool for making treatment decisions in daily practice.

Recently, deep learning-based semantic segmentation has been developed and used in diverse medical fields, especially for image analysis. There have been many previous reports on semantic segmentation of OCT images, but most studies have focused on how to establish methods and their accuracy relying on segmental methods [2,3,4,5,6], while studies on clinical changes in DME and their correlates are relatively scarce, with varied artificial intelligence (AI) findings between studies. In addition, most AI-based studies investigating diseases that cause macular fluid (MF) have typically used whole OCT images to predict relationships between retinal morphology and visual acuity [7] as well as their response to anti-vascular endothelial growth factor (VEGF) drug therapy [3,8]. It is known that ellipsoid zone (EZ) loss correlates with visual acuity decrease in DME, and typical AI studies using whole OCT images have inevitably shown that EZ disruption correlates more strongly with visual acuity than intraretinal cystoid fluid and the central subfield thickness [4]. However, it is quite possible that prolonged retention of MF may cause EZ loss and subsequent reduction in visual acuity. This suggests that AI analysis using whole OCT images may not accurately evaluate the impact of MF on visual acuity, because it focuses largely on EZ structures.

In this study, we examined changes in qualitative and quantitative MF parameters with the progression of DR stages and their relationships with visual acuity using mask-processed OCT images in which only MF areas of DME were extracted.

## 2. Materials and Methods

### 2.1. Study Subjects

This is a retrospective observational case study. This study followed the principles of the Declaration of Helsinki. The institutional review board in Hokkaido University Hospital (IRB number: C-T2023-0331) approved this study. The IRB at Hokkaido University Hospital approved the study on an opt-out basis, in which patients were given the opportunity to refuse to participate in the study via the website since this is a non-invasive retrospective observational study. A total of 104 eyes of 71 Japanese patients (64.2 ± 10.2 years of age, 44 males with 64 eyes, 27 females with 40 eyes) who were diagnosed with treatment-naïve DME at our institution between January 2010 and April 2023 were enrolled in this study. DME was diagnosed based on the first observation of IRF, SRF, or both in the central fovea on OCT horizontal sections during the course of DR.

All participants underwent a medical history inquiry, comprehensive clinical examinations, and ophthalmic examinations, including BCVA, intraocular pressure, slit-lamp microscopy, fundus examinations, and ocular imaging. The horizontal scans of swept-source OCT (DRI OCT Triton or DRI OCT-1 Atlantis; Topcon Inc., Tokyo, Japan) imaged with a single-line B-scan or spectral-domain OCT (RS-3000 or RS-3000 Advance; NIDEK, Gamagori, Japan) imaged with five cross-line B-scans, centered on the fovea at the time when DME was first observed, was used to detect MF. The clearest image, with signal-to-noise ratios above 8 and eligible for AI analysis among all B-scans obtained from two or three imaging sessions per patient, was used for MF segmentation. BCVA data were collected from the medical record on the same day as the OCT images were taken, and the decimal BVCA was converted into logMAR BCVA. DME eyes were classified into mNPDR, sNPDR, and PDR by one of authors (M.M.) based on medical records or fundus photographs. A semantic segmentation technique was used to identify the sites on the OCT images of IRF and SRF separately, and the areas were subsequently measured.

The exclusion criteria were as follows: (1) patients having a history of previous topical drug treatments for DME including intravitreal anti-VEGF injections and/or periocular/intravitreal triamcinolone acetonide injections, and (2) patients with macular edema due to other retinal diseases including retinal vein occlusion, neovascular age-related macular degeneration, and idiopathic macular telangiectasia.

### 2.2. The Neural Network for MF Segmentation

Recently, we developed a semantic segmentation model named the “HUMFS model” [9] using modified deep U-net architecture with trained MF data, which generates predictive images of MF by semantic segmentation from DME OCT images using NNabla version 1.33.1, (Sony Corporation, Tokyo, Japan). Four-fifths of the 104 images were used for the training and one-fifth was used for validation of the “HUMFS model”. The training error was 0.004668, the validation error was 0.014386, and the learning curves for both converged and showed no tendency toward overlearning [9]. The performance of MF segmentation was assessed using images from the validation dataset and the validation data were found to have a sensitivity of 0.767, a specificity of 0.996, an accuracy of 0.993, an area under the curve of 0.986, and a Dice coefficient of 0.702 [9]. In the present study, we used the “HUMFS model” modified to identify IRF and SRF separately.

### 2.3. Generation of MF Images in a Representative Case

**Figure 1:**
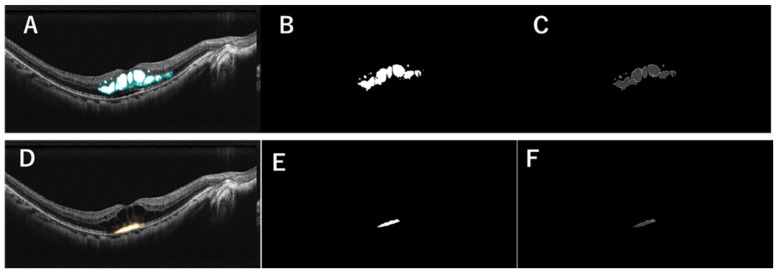
The representative macular fluid images of the mild or moderate non-proliferative diabetic retinopathy case. (**A**) Intraretinal fluid (IRF) recognized by artificial intelligence (AI). (**B**) Binarized image for measuring IRF area. (**C**) Image weighted to measure the mean brightness, and variance of brightness. (**D**) Subretinal fluid (SRF) recognized by AI. (**E**) Binarized image for measuring SRF area. (**F**) Image weighted to measure the mean brightness, and variance of brightness.

Figure 1 shows representative images for a generation of MF images in this study. AI recognized and divided MF into IRF (Figure 1A) and SRF (Figure 1D), binarized the images (Figure 1B,E) and measured the area (pixels) of IRF and SRF. Furthermore, the mean brightness and the variance of brightness (no unit for both) of IRF and SRF were measured on mask-processed OCT images, i.e., images in which only MF areas were extracted by using the images created by the “HUMFS model” (Figure 1C,F).

### 2.4. Endpoints and Statistical Analyses

The primary endpoints were the area, the mean brightness, and the variance of brightness of IRF and SRF in DME eyes at each stage of mNPDR, sNPDR, and PDR. The area was chosen as a quantitative indicator and the mean brightness and the variance of brightness as qualitative indicators to reveal changes in the microstructure of MFs during the DR progression. Next, correlations between logMAR BCVA and MF parameters (the area, the mean brightness, and the variance of brightness) were examined for all DME eyes.

Statistical tests were performed using the R statistical package (version 3.6.1, R Foundation for Statistical Computing, Vienna, Austria). The Kruskal–Wallis test and post hoc Steel-Dwass test were used to compare changes in age, logMAR BCVA, the area, the mean brightness, and the variance of brightness of IRF and SRF among mNPDR, sNPDR, and PDR. The Chi-square test and post hoc Fisher’s exact test with Holm’s correction were used to analyze correlations in the rates of pseudophakic eyes, pan-retinal photocoagulation, and complication of ERM among mNPDR, sNPDR, and PDR. Spearman’s rank correlation coefficient was used to analyze correlations between logMAR BCVA and the MF parameters, given that normality of distribution was rejected. A *p*-value < 0.05 was determined as statistically significant. Data were presented as mean ± standard deviation.

## 3. Results

### 3.1. Clinical Background

A total of 33, 52, and 19 eyes were enrolled in mild or moderate non-proliferative DR (mNPDR), severe NPDR (sNPDR), and proliferative DR (PDR) groups, respectively. As shown in Table 1, patients with PDR eyes were younger than those with mNPDR eyes (*p* < 0.01) and sNPDR eyes (*p* < 0.05). The logMAR best-corrected visual acuity (BCVA) of PDR eyes was significantly higher than that of mNPDR eyes (*p* < 0.01) and sNPDR eyes (*p* = 0.020), which was acceptable since all DME eyes enrolled in this study were treatment-naïve. There were no significant differences in the ratio of pseudophakic eyes (*p* = 0.058) or complication of ERM (*p* = 0.35). The ratio of eyes receiving pan-retinal photocoagulation was higher in sNPDR and PDR eyes than in mNPDR eyes (*p* < 0.001).

### 3.2. Changes in the MF Parameters Depending on DR Stages

**Figure 2:**
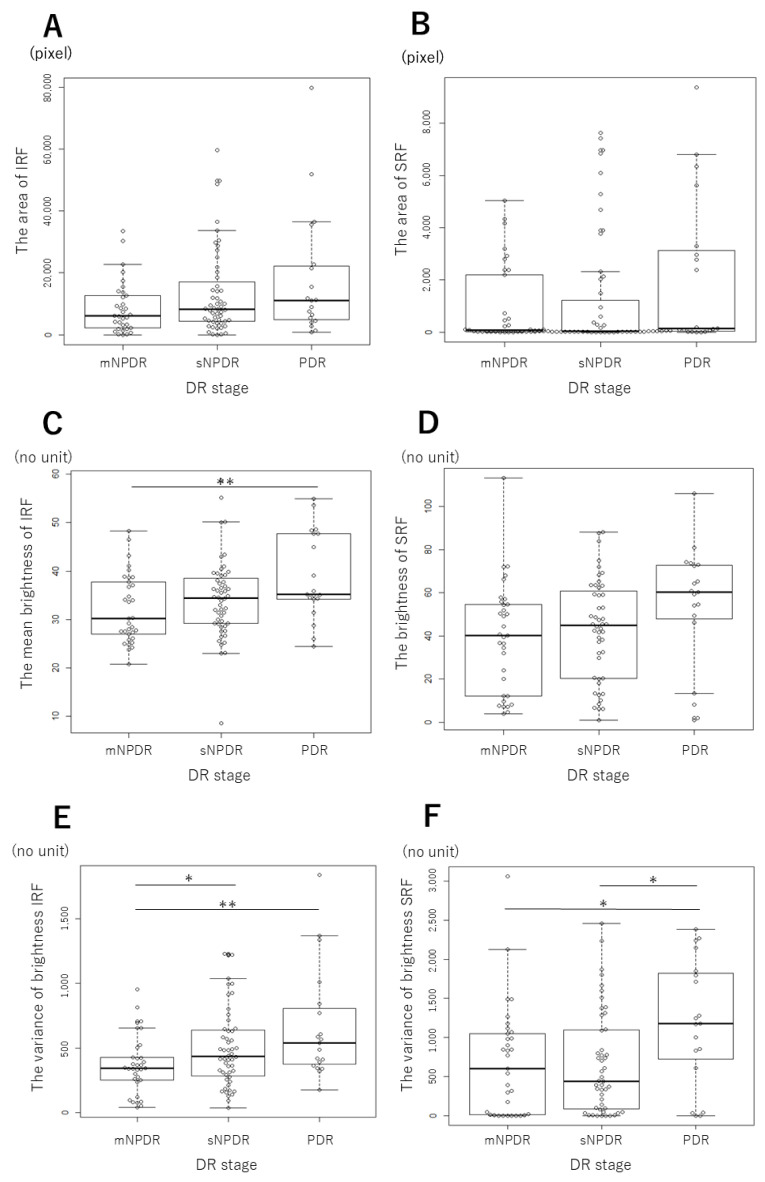
The macular fluid parameters at each diabetic retinopathy stage. (**A**) The IRF area. (**B**) The SRF area. (**C**) The mean brightness of IRF. (**D**) The mean brightness of SRF. (**E**) The variance of brightness of IRF. (**F**) The variance of brightness of SRF. Intraretinal fluid, IRF; subretinal fluid, SRF; mild or moderate non proliferative diabetic retinopathy, mNPDR; severe NPDR, sNPDR; proliferative diabetic retinopathy, PDR. * *p* < 0.05, ** *p* < 0.01, Kruskal–Wallis test, post hoc Steel-Dwass test.

For mNPDR, sNPDR, and PDR eyes, the MF area was 8747 ± 8486, 13,135 ± 13,629, and 17,825 ± 20,348 pixels for IRF (*p* = 0.17) (Figure 2A), and 968 ± 1524, 1225 ± 2278, and 2118 ± 2921 pixels for SRF (*p* = 0.27) (Figure 2B). The IRF area tended to increase with the progression of DR with statistically insignificant differences. The SRF area showed no statistically significant differences depending on DR stages.

The mean brightness was 32.1 ± 7.1, 34.3 ± 8.3, and 38.9 ± 9.1 for IRF (*p* < 0.05) (Figure 2C), and 39.2 ± 25.4, 42.8 ± 23.4, and 53.6 ± 28.5 for SRF (*p* = 0.057) (Figure 2D). The mean brightness of IRF significantly increased in PDR compared with mNPDR (post hoc, *p* < 0.01). The mean brightness of SRF tended to increase with the progression of DR with statistically insignificant differences depending on DR stages.

The variance of brightness was 370 ± 226, 499 ± 304, and 670 ± 435 for IRF (*p* < 0.05) (Figure 2E), and 679 ± 710, 662 ± 657, and 1192 ± 810 for SRF (*p* < 0.05) (Figure 2F). The variance of brightness of IRF was significantly greater in sNPDR and PDR than in mNPDR (post hoc, *p* < 0.05 for both). The variance of brightness of SRF was significantly greater in PDR than in mNPDR and sNPDR (post hoc, *p* < 0.05 for both). Overall, the variance of brightness of IRF increased in sNPDR compared to mNPDR, and remained unchanged thereafter, while the variance of brightness of SRF stayed unaltered from mNPDR to sNPDR and then increased in PDR compared to sNPDR.

### 3.3. Correlations Between the MF Parameters and logMAR BCVA

**Figure 3:**
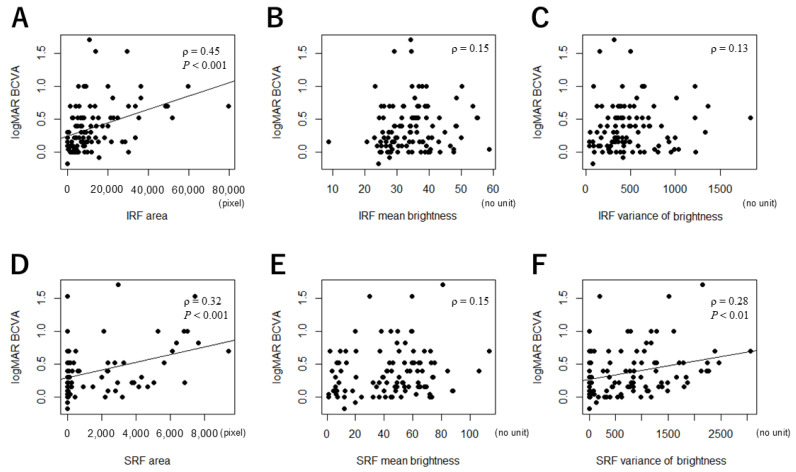
The correlation between logMAR BCVA and macular fluid parameters. (**A**) Correlation between logMAR BCVA and the IRF area. (**B**) LogMAR BCVA and the mean brightness of IRF. (**C**) LogMAR BCVA and the variance of brightness of IRF. (**D**) LogMAR BCVA and the SRF area. (**E**) LogMAR BCVA and the mean brightness of SRF. (**F**) LogMAR BCVA and the variance of brightness of SRF. Best-corrected visual acuity, BCVA; Intraretinal fluid, IRF; subretinal fluid, SRF. Spearman’s rank correlation coefficient.

Next, we examined correlations between the MF parameters and logMAR BCVA for all DME eyes. LogMAR BCVA had a statistically weak positive correlation with the IRF area (ρ = 0.45, *p* < 0.001) (Figure 3A), the SRF area (ρ = 0.32, *p* < 0.001) (Figure 3D), and the variance of brightness of SRF (ρ = 0.28, *p* < 0.01) (Figure 3F). There were no significant correlations between logMAR BCVA and the following MF parameters: the mean brightness of IRF (ρ = 0.15, *p* = 0.12) (Figure 3B), the variance of brightness of IRF (ρ = 0.13, *p* = 0.21) (Figure 3C), and the mean brightness of SRF (ρ = 0.15, *p* = 0.17) (Figure 3E).

## 4. Discussion

We assessed the multiple aspects of MF in DME eyes in not only quantitative terms (i.e., cumulative amount) but also qualitative (i.e., internal properties) parameters based on the “Hokkaido University macular fluid segmentation model” (HUMFS model) [9], which enables numerical evaluations. This technique revealed several important findings as follows: (1) The IRF area tended to increase with progression of DR, although there were no statistically significant differences in the SRF area between DR stages; (2) the mean brightness of IRF and SRF tended to increase depending on DR stages; (3) the variance of brightness of IRF increased in sNPDR eyes compared with mNPDR eyes, while the variance of brightness of SRF remained unchanged from mNPDR to sNPDR and then increased in PDR, showing a difference in the timing of their increases during the course of DR progression; and (4) logMAR BCVA had statistically weak positive correlations with IRF area, SRF area, and the variance of brightness of SRF.

In past investigations, qualitative parameters of MF included the morphologic and regional features of OCT (e.g., spongiform or cystoid, and IRF or SRF) and the presence or absence of associated findings such as hyperreflective foci and fibrinogen clots, whereas quantitative parameters of MF include the area or volume of MF and retinal thickness. To the best of our knowledge, there have been no reports on the numerical evaluation of qualitative MF parameters depending on DR stages. In this study, we successfully made numerical evaluations for both the mean brightness and the variance of brightness as qualitative parameters, as well as the MF areas as a quantitative parameter using our AI semantic segmentation model.

In this study, the IRF area tended to increase with the progression of DR with statistically insignificant differences. Although the prevalence of DME generally increases with the progression of DR stages, DME can develop from the early stage of DR [10], as a result of serous leakage from microaneurysms, one of the earliest signs of DR [11,12]. The mildly increasing trend in the IRF area could be explained by the following mechanisms in IRF accumulation: serious leakage due to microaneurysms, which are common throughout DR stages, and increased leakage dependent on ischemia-induced upregulation of VEGF production, which is more strongly related to DR progression. On the other hand, the SRF area failed to show statistically significant differences between DR stages. This is partly because of the small number of SRF-positive cases; SRF was observed in only 42% of cases, whereas IRF was observed in all cases. As for pathological explanations for SRF accumulation, the primary process leading to the development of SRF is the influx of IRF into the subretinal space following disruption of external limiting membrane [13]. Moreover, the choroidal vascular changes associated with DR may also contribute to the accumulation of SRF, which is known as diabetic choroidopathy characterized by increased choroidal thickness in the relatively late stage of DR [14]. This choroidal thickening is considered inflammatory swelling and is implicated in secondary SRF exudation. Therefore, the development of SRF requires more complex and diverse pathological conditions than IRF, leading to the small number of less than half the patients with DME. In this study, both MF areas proved to be of limited importance as parameters to reflect DR severity.

In contrast, the qualitative MF parameters, the mean brightness and the variance of brightness, showed significant increases with the progression of DR stages. Under physiological conditions, a tight junction constitutes the inner blood–retinal barrier (BRB), and proteins cannot pass through the retinal vessel wall [13]. However, under DM conditions, oxidative stress induced by glucose toxicity activates endothelial cells. Subsequently, VEGF and inflammatory cytokines, including monocyte chemotactic protein-1 and interleukin-6 [13], cause tight junction dysfunction, resulting in microvascular damage and subsequent leakage of high-molecular weight (HMW) proteins such as albumin and fibrinogen, which are normally unable to pass through the vessel walls. The internal brightness of DME reflects the concentration of these HMW proteins as highly reflective materials [15,16,17]. In addition, a soluble form of fibrinogen develops into fibrin clots within the cyst and contains hardly soluble substances such as advanced glycation end-products [18]; the aggregation of these materials is detected as the high variance of brightness in MF. In the current study, the qualitative changes, including leakage of the HMW proteins from the damaged vessel wall and deposition of hardly soluble substances, were sensitively detected as the stage-by-stage increases in the mean brightness and the variance of brightness.

Interestingly, the variance of brightness of IRF increased in sNPDR, while the variance of brightness of SRF increased in PDR with statistical significances, showing a difference in the timing of their increases during the course of DR progression. This may be due to the different mechanisms between IRF and SRF. DR is primarily a retinal vascular endothelial cell disorder causing IRF, and retinal pigment epithelial cells are secondarily damaged by overload to overcome excessive SRF retention. IRF is only attributed to the disruption of the inner BRB, while SRF is affected by choroid-derived fluid influx due to disruption of the outer BRB [14] associated with diabetic choroidopathy [13]. The complexity of the SRF pathophysiology may have led to the delayed increase in the variance of brightness of SRF compared with that of IRF.

In this study, logMAR BCVA had statistically weak positive correlations with the IRF area, SRF area, and the variance of brightness of SRF. There were reports that baseline visual acuity was correlated with macular volume [19,20,21], consistent with the IRF area results in our present study. The IRF area may correlate with visual acuity due to retinal structural damage caused by retinal deformation with elongation of cellular elements secondary to the presence of cystoid spaces [19]. SRF was reported to be associated with deteriorated logMAR BCVA [22], suggesting that the detrimental proteins in SRF is a predisposing factor in photoreceptor damage. In our study, there were cases with small MF areas but poor visual acuity, suggesting that factors other than MF, including damage to outer retinal layers, may have caused visual loss.

This study has some limitations. First, this is a retrospective observational study with a relatively small number of cases. To further validate our results showing the stage-dependent changes in DME, a longitudinal cohort study is warranted. Second, accurately identifying MF on OCT is sometimes a task so difficult for humans as to negatively influence the generation of teaching data. Therefore, the IRF and SRF identified by our AI model could not necessarily detect MF precisely. In addition, there is currently no established method to fully correct the variation in brightness of OCT images among different scans from different eyes. To further validate the HUMFS model, it would be desirable to include inter-rater reliability data in manual segmentation and to establish an image brightness correction analysis.

In conclusion, we analyzed quantitative and qualitative MF parameters of DME using AI. The qualitative parameters, the mean brightness and the variance of brightness, were more strongly related to DR stages than the quantitative parameter. The qualitative MF parameters may reflect changes in the concentration and heterogeneity of highly reflective materials and may be useful not only to better understand DME pathogenesis according to DR progression, but also to complement patient risk stratification and treatment decisions.

## Figures and Tables

**Table 1:** Clinical characteristics in mNPDR, sNPDR, and PDR groups.

	mNPDR (n = 33)	sNPDR (n = 52)	PDR (n = 19)	*p* Value			
				All the 3 Groups	mNPDR vs. sNPDR	mNPDR vs. PDR	sNPDR vs. PDR
Age (years)	66.3 ± 9.6	64.5 ± 11.3	59.8 ± 6.0	**<0.01** ^☨^	0.90 ^☨☨^	**<0.01** ^☨☨^	**<0.05** ^☨☨^
logMAR BCVA	0.27 ± 0.29	0.37 ± 0.37	0.57 ± 0.36	**<0.01** ^☨^	0.41 ^☨☨^	**<0.01** ^☨☨^	**<0.05** ^☨☨^
Pseudophakic eye (%)	15 (45.5%)	14 (26.9%)	3 (15.8%)	0.058 ^#^	NA	NA	NA
PRP (%)	3 (9.1%)	25 (48.1%)	7 (36.8%)	**<0.001** ^#^	**<0.001** ^##^	0.052 ^##^	0.43 ^##^
ERM (%)	6 (18.2%)	10 (19.2%)	1 (5.2%)	0.35 ^#^	NA	NA	NA

mNPDR, mild or moderate non-proliferative diabetic retinopathy; sNPDR, severe non-proliferative diabetic retinopathy; PDR, proliferative DR; BCVA, best-corrected visual acuity; PRP, pan-retinal photocoagulation; ERM, epiretinal membrane; NA, not applicable. ☨ Kruskal–Wallis test; ☨☨ post hoc Steel-Dwass test, # chi-square test, ## post hoc Fisher’s exact test with Holm’s correction. Statistically significant values are highlighted as bold.

## Data Availability

The data that support the findings of this study are available on request from the corresponding author, M.M. and M.S. The data are not publicly available due to their containing information that could compromise the privacy of research participants.
